# The complete mitochondrial genome sequence of the corn planthopper, *Peregrinus maidis* (Hemiptera: Fulgoroidea)

**DOI:** 10.1080/23802359.2017.1398605

**Published:** 2017-11-07

**Authors:** Yi-Xin Huang, Dao-Zheng Qin

**Affiliations:** Key Laboratory of Plant Protection Resources and Pest Management, Ministry of Education, Entomological Museum, Northwest A&F University, Yangling, Shaanxi, China

**Keywords:** Delphacidae, mtDNA, phylogenetic relationship

## Abstract

In this study, we analyzed the complete mitochondrial genome sequence of the corn planthopper, *Peregrinus maidis*. The complete mitogenome sequence of *P. maidis* was observed to be a circular molecule 16,279 bp long and consisting of 13 protein-coding genes (PCG), 2 ribosomal RNA (rRNA) genes and 22 transfer RNA (tRNA) genes (GenBank accession no. MG049917). The nucleotide composition is biased toward adenine and thymine (77.8% A + T). The A + T-rich region was found between *rrnS* and *trnI*, and this entire region was 1596 bp long.

The corn planthopper, *Peregrinus maidis* (Ashmead 1890) is a widely distributed destructive insect that causes significant yield losses not only by feeding on vascular tissues via piercing-sucking mouthparts, but also by transmitting viruses between crops (Lastra and Esparza 1976; Nault and Ammar 1989; Yao et al. 2013). Despite its economic importance, the mitogenome sequence of *P. maidis* so far remained unknown. Therefore, we sequenced the complete mitochondrial DNA genome of *P. maidis* to provide more comprehensive data for this species and also for its relationship within the family Delphacidae.

Adult *P. maidis* males were collected from a maize field in Xiashi Town (N 22°07′17.39″ and E 106°54′0.89″), Guangxi, China, in August 2014. Voucher specimens were deposited in the Key Laboratory of Plant Protection Resources and Pest Management of Ministry of Education, Entomological Museum, Northwest A&F University (NWAFU). The complete mitochondrial genome of *P. maidis* was determined by using next-generation sequencing (NGS).

The *P. maidis* mitochondrial genome is 16,279 bp (Genbank accession no. MG049917) in length with a total A + T content of 77.8% that is heavily biased toward the A and T nucleotides. It encodes the complete set of 37 genes which are usually found in animal mitogenomes. The gene arrangement in the mitochondrial genome of *P. maidis* is conserved, similar to other mitogenomes in Delphacidae, with the exception of *Nilaparvata lugens* (Zhang et al. 2013). In the mitogenome of *P. maidis*, a total of 19 bp overlaps have been found at nine gene junctions (*trnQ* and *trnM* share a nucleotide; *nad2* and *trnC* share two nucleotides; *atp8* and *atp6* share four nucleotides; *trnR* and *trnN*, *trnN* and *trnS_1_*, *trnS_1_* and *trnE*, *trnE* and *trnF* share one nucleotide; *nad4* and *nad4L* share seven nucleotides; and *cytb* and *trnS_2_* share one nucleotide). The mitogenome is loose and has a total of 488 bp intergenic sequences without the putative A + T-rich region. The intergenic sequences are at 15 locations ranging from 1 to 338 bp, with the longest one located between *trnP* and *nad6*. The A + T-rich region of the *P. maidis* is 1596 bp long and located between the *rrnS* and *trnI* genes. The A + T content of this region is 86.2%.

All 22 tRNA genes usually found in the mitogenomes of insects are present in *P. maidis*. The nucleotide length of tRNA genes ranges from 55 bp (*trnH*) to 71 bp (*trnK*), and A + T content ranges from 70.4% (*trnK*) to 89.3% (*trnS_2_*). These two rRNA genes have been identified on the N-strand in the *P. maidis* mitogenome.

We analyzed the nucleotide sequences of 13 PCGs using the maximum likelihood (ML) method to understand the phylogenetic relationship of *P. maidis* with other fulgoroids. The mitogenome sequence of *Hyalessa maculaticollis* (GenBank accession no. JQ910987) was used as outgroup. The result shows that *P. maidis* belongs to the superfamily Fulgoroidea and is clustered into a branch of Delphacidae (Figure 1). The family Delphacidae is monophyletic and *P. maidis* is supported as the sister group to the remaining delphacids.

**Figure 1.**
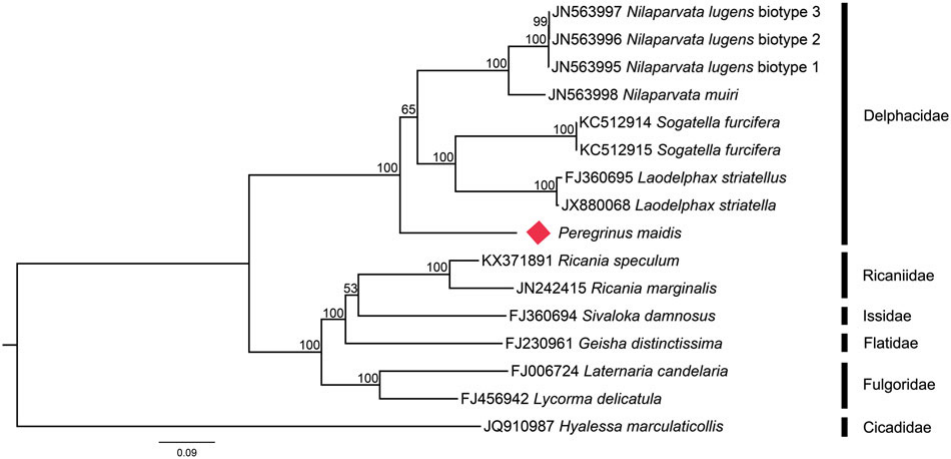
The maximum-likelihood (ML) phylogenetic tree of *P. maidis* and other fulgorids. The numbers beside the nodes are percentages of 1000 bootstrap values. Alphanumeric terms indicate the GenBank accession numbers.
